# Diagnostic Approaches for Measles Virus: Methods, Advances, and Ongoing Challenges

**DOI:** 10.3390/pathogens14121295

**Published:** 2025-12-17

**Authors:** Yuan-Chao Xue, Ping Ren

**Affiliations:** Department of Pathology, University of Texas Medical Branch, Galveston, TX 77555, USA; yuaxue@utmb.edu

**Keywords:** measles virus, laboratory diagnostics, clinical microbiology

## Abstract

Measles, also known as rubeola, is a highly contagious and potentially life-threatening disease caused by the measles virus. It classically presents with fever, cough, coryza, conjunctivitis, and a maculopapular rash. Despite the availability of an effective vaccine for decades, measles outbreaks continue to occur globally, largely driven by declining vaccination coverage and increased international travel. With no specific antiviral therapy available, rapid and accurate diagnosis remains essential for timely clinical management and effective outbreak control. Diagnostic methods have evolved from traditional virus isolation in cell culture to serologic assays and, more recently, to molecular techniques such as real-time reverse transcriptase polymerase chain reaction (rRT-PCR). Each diagnostic method has unique advantages and limitations influenced by specimen type, timing of collection, and laboratory capacity. This minireview summarizes the progress of measles virus diagnostics, outlines current laboratory detection strategies, and discusses emerging technologies and ongoing challenges amid global measles resurgence and increasing public health demands.

## 1. Introduction

Measles virus (MeV), *Morbillivirus hominis*, a member of the *Morbillivirus* genus within the *Paramyxoviridae* family, is an enveloped, pleomorphic virus containing a single-stranded, non-segmented, negative-sense RNA genome approximately 16 kb in length [1]. The genome comprises six genes encoding eight proteins. Viral particles typically range from 100 to 250 nm in diameter. Although MeV is classified as a single serotype, antigenic and genotypic variations exist among circulating wild-type strains and between wild-type and vaccine-derived viruses [1].

MeV remains one of the most contagious human pathogens, and global transmission has increased in recent years. This resurgence is driven primarily by disruptions to routine immunization programs during the COVID-19 pandemic and by the growing vaccine hesitancy in many regions [2]. In this context, rapid and accurate detection of MeV is critical for timely clinical management, implementation of infection prevention measures, and effective outbreak containment.

## 2. Clinical Symptoms, Vaccination and Management

Measles-related symptoms typically develop 10–14 days after infection and begin with a prodromal phase characterized by fever, cough, coryza, and/or conjunctivitis lasting 2–4 days [2]. Koplik spots, small white lesions on the buccal mucosa, may appear 1–2 days before rash onset and can persist for 1–2 days afterward. The classic measles rash is an erythematous, maculopapular exanthem that emerges 2–4 days after fever onset, starting on the face and spreading caudally to the head, trunk, and extremities. Patients are contagious from 4 days before to 4 days after the appearance of the rash [2]. Clinically, the combination of prodromal symptoms with a descending maculopapular rash in a child, sometimes accompanied by diarrhea, should raise a strong suspicion for MeV infection.

MeV infection can involve multiple organ systems, including the skin, eyes, respiratory tract, and gastrointestinal tract [2]. As a result, complications occur in approximately 30% of cases within the first month of illness and commonly include diarrhea, pneumonia, otitis media, and conjunctivitis. Measles-associated encephalitis, although uncommon, is one of the most severe complications and can occur within the first week of illness, between 1–6 months after infection, or even years later as subacute sclerosing panencephalitis (SSPE) following an apparently complete recovery [2,3].

Live attenuated measles vaccines are widely used and remain the cornerstone of global measles prevention [2]. Vaccination is particularly critical for young children, who are at the highest risk for severe disease. Recommended timing of the first dose varies by epidemiologic setting: in regions where measles has been eliminated, the first dose is administered at 12–18 months of age, whereas in endemic areas, vaccination at 9 months is advised due to earlier exposure risk and waning maternal antibodies [2]. If seroconversion does not occur or the first dose is missed, an additional dose should be given after 9 months of age and at least 4 weeks after the initial dose, regardless of geographic region.

There are currently no approved antiviral therapies for MeV infection [2]. For postexposure prophylaxis, measles vaccination is recommended within 72 h of exposure. However, it is contraindicated in individuals who are immunocompromised, pregnant, or younger than 6 months of age [2]. In such cases, human immune globulin may be administered within 6 days of exposure, although its use is limited by cost and availability. As a result, effective measles management relies heavily on early recognition and laboratory confirmation of MeV infection to guide timely clinical decision-making, isolation precautions, and public health interventions.

## 3. Current Diagnostic Methods

### 3.1. Virus Isolation

Isolation of MeV in cell culture, typically using Vero/hSLAM cells, is now rarely performed for routine diagnosis [1,4]. Instead, it is reserved for research, molecular epidemiology, and outbreak investigations where high viral titers or comprehensive viral characterization is needed. Optimal isolation depends on the collection of respiratory specimens, urine, or peripheral blood mononuclear cells within 3–5 days of rash onset and processing under standard biosafety level 2 (BSL-2) conditions [1,4]. Confirmation is typically achieved using real-time reverse transcriptase polymerase chain reaction (rRT-PCR), immunofluorescence, or immunohistochemistry. Although highly specific, the slow turnaround time and limited sensitivity in late infection restrict its diagnostic utility.

### 3.2. Serology

Two primary platforms, enzyme immunoassay (EIA) and chemiluminescent immunoassay (CLIA), are used for serologic detection of MeV infection [1]. EIAs rely on enzyme-mediated colorimetric detection, while CLIAs measure antibody-antigen binding via luminescence and offer improved throughput and automation [4]. Both approaches commonly target the MeV nucleocapsid (N) protein, a highly abundant antigen that elicits a strong IgM response. However, a meta-analysis has demonstrated substantial variability in sensitivity and specificity among commercial assays, influenced by patient age, gender, vaccination status, geography, and timing of sample collection [5].

Detection of measles-specific IgM remains the serologic standard for confirming acute infection [5]. Current World Health Organization (WHO) and U.S. Centers for Disease Control and Prevention (US CDC) guidelines recommend dual testing with IgM serology and rRT-PCR to maximize diagnostic accuracy [6,7]. Serum for IgM testing should be collected shortly after rash onset [6]. However, in previously vaccinated individuals, breakthrough infections often generate attenuated, delayed, transient, or even absent IgM production despite true infection [8]. This substantially reduces the sensitivity and positive predictive value of IgM assays in highly vaccinated populations.

In these breakthrough cases, rRT-PCR generally retains greater diagnostic sensitivity because MeV RNA can be detected even when IgM responses are blunted. Nevertheless, viral loads may be lower and clear more rapidly than in primary infections, which narrows the window of PCR detection and can lead to false-negative results if specimens are not collected promptly or from optimal sites [4]. When IgM is negative and rRT-PCR is inconclusive, serologic assessment of measles-specific IgG in paired acute and convalescent sera provides an important complementary strategy. A ≥4-fold rise in IgG titers supports recent infection and is particularly valuable when nucleic acid detection is negative despite strong clinical suspicion [1].

MeV-specific IgM typically wanes within 6–8 weeks, while IgG appears shortly after IgM and persists for life [1]. Demonstration of seroconversion or a ≥4-fold rise in MeV-specific IgG titers in paired acute and convalescent sera (taken 10–14 days apart) can confirm recent infection, particularly in unvaccinated or under vaccinated individuals. IgG avidity testing also aids interpretation: low avidity indicates recent infection, while high avidity supports past infection or vaccination [1]. Additionally, detection of MeV-specific IgG in both serum and cerebrospinal fluid (CSF), indicating intrathecal antibody production, is useful in diagnosing measles encephalitis and subacute sclerosing panencephalitis [1].

In regions where measles has been eliminated, the reduced positive predictive value of IgM underscores the importance of confirmatory nucleic acid testing and, when necessary, paired serology for accurate case classification [1].

### 3.3. Plaque Reduction Neutralization

The plaque reduction neutralization test (PRNT) is the reference standard for quantifying measles-neutralizing antibodies [4]. Despite its accuracy, PRNT is labor-intensive and requires BSL-2 facilities, fresh monkey erythrocytes, extensive hands-on time, and 5–7 days to complete [4,9]. These limitations restrict its use to specialized or research laboratories.

The focus reduction neutralization test (FRNT) was developed to overcome PRNT’s limitations [4,10,11]. FRNT offers reduced turnaround time (2–3 days), smaller sample volume requirements, colorimetric readout, and compatibility with automation [4,10,11]. Importantly, FRNT measures neutralizing antibody that correlates with PRNT results, making it a practical alternative for large-scale seroepidemiologic studies.

### 3.4. Real-Time Reverse Transcriptase-Polymerase Chain Reaction

The US CDC recommends rRT-PCR as the primary method for confirming MeV infection [6]. It offers rapid turnaround time, high analytical sensitivity, reduced contamination risk, and quantitative capability [1,4,12]. Assays can be tailored to distinguish wild-type MeV from vaccine-derived genotype A strains, which is critical during outbreak investigations in highly vaccinated populations [13,14]. Some multiplex PCR panels can also detect alternative etiologies of febrile rash illnesses, aiding differential diagnosis [4,15].

MeV RNA can be detected in respiratory and urine specimens for up to 14 days after rash onset, and the high sensitivity of rRT-PCR allows detection of low viral loads, including in breakthrough infections [1]. Nevertheless, despite MeV’s relatively low genetic variability, primer and probe mismatches can impact assay performance, reinforcing the need for ongoing genomic surveillance and periodic assay updates [13,16]. Additionally, diagnostic accuracy also depends on appropriate specimen type, timing of collection, and transport and storage conditions [4].

In response to a recent measles cluster in West Texas, our laboratory validated an automated laboratory-developed test (LDT) to enhance local diagnostic capacity. This assay employed revised US CDC MeV primers and probes [17] and was performed on nasopharyngeal specimens collected in Universal Transport Medium (UTM) using the Hologic Panther Fusion Open Access platform (Marlborough, MA). The LDT achieved a limit of detection (LOD) of approximately 0.125 plaque-forming units/mL and demonstrated 100% analytical sensitivity (95% confidence interval (CI): 92.89–100%) and 100% analytical specificity (95% CI: 97.45–100%). A manual rRT-PCR assay performed on extracted or naked nucleic acid preparations showed an LOD of approximately 33 RNA copies/mL [17]. These findings highlight the potential of automated, sample-to-answer platforms to streamline workflows without compromising diagnostic sensitivity, particularly during outbreak settings.

### 3.5. Rapid Diagnostic Tests

Lateral flow-based rapid diagnostic tests (RDTs) provide a practical option for decentralized or resource-limited settings, requiring no specialized instrumentation, refrigeration, or extensive training [4,18]. RDTs rely on capillary flow and antigen–antibody binding kinetics, consisting of four main components: a sample pad, a conjugate pad with gold nanoparticle-conjugated antibodies, a nitrocellulose membrane with immobilized capture antibodies, and an absorbent pad [19]. Most assays target the MeV N protein and deliver results within 30 min [4]. Although less analytically sensitive than EIA, viral RNA can be extracted from RDT membranes for downstream molecular analysis, including rRT-PCR or sequencing [20].

RDTs expand diagnostic capacity in settings lacking laboratory infrastructure and improve surveillance by enabling point-of-care specimen collection [18]. Positive or inconclusive RDT results can be forwarded to national or regional reference laboratories for confirmation using EIA, rRT-PCR, or sequencing. Reference laboratories also play a role in the quality management and assurance of RDTs implementation [18]. However, challenges remain, including the need for comprehensive validation, regulatory approval pathways, and sustainable funding models to support long-term development.

## 4. Emerging Diagnostic Platforms

### 4.1. Digital Droplet Polymerase Chain Reactions

Digital droplet polymerase chain reaction (ddPCR) is an emerging molecular technology that provides direct, absolute quantification of nucleic acids, including viral RNA, with exceptional precision, without the need for standard curves or external calibrators [4,21,22]. The method partitions DNA or cDNA into thousands of nanoliter-sized droplets before amplification, allowing fluorescence detection in individual droplets and Poisson-based quantification of target copy numbers [4,21,22].

For measles diagnosis, ddPCR offers several potential advantages. ddPCR allows for precise and direct quantification of nucleic acids, removing the need for standard curves or reference controls. This eliminates the variability and complexity that comes with relative quantification. Its superior analytical sensitivity enables detection of low-level MeV RNA, which may be particularly valuable in cases with minimal viral shedding, such as modified or breakthrough infections in vaccinated individuals. The platform’s tolerance to PCR inhibitors also supports its use in nontraditional or challenging matrices, including wastewater, where measles surveillance is increasingly being explored [23,24,25,26,27]. In addition, ddPCR maintains the multiplexing flexibility of traditional PCR, allowing simultaneous detection of MeV together with other rash-causing viral pathogens such as rubella [4,21,22].

Despite its promise, ddPCR is not routinely used for clinical MeV diagnostics. Barriers include high upfront equipment costs, limited availability of commercial MeV-specific assay, more complex workflows, and the requirement for specialized technical expertise [4]. Importantly, comprehensive clinical validation studies and standardized operating protocols remain limited, and broader adoption will depend on the development of harmonized assays and clearer clinical use cases.

### 4.2. Next-Generation Sequencing

Next-generation sequencing (NGS) provides high-resolution genomic characterization and is increasingly employed in infectious diagnostics, epidemiology, and public health surveillance [28]. Common NGS strategies include (i) amplicon-based sequencing using overlapping PCR products, (ii) targeted enrichment with biotinylated oligonucleotide probes to enhance sensitivity, and (iii) untargeted shotgun metagenomic sequencing directly from clinical specimens [28,29].

NGS is particularly well-suited to address challenges associated with global measles resurgence. Full-genome sequencing of MeV enables detailed molecular epidemiology, including outbreak reconstruction, transmission chain mapping, identification of importations, and differentiation of wild-type from vaccine-derived strains, critical for surveillance in highly vaccinated populations [4,30,31]. During outbreaks, NGS can complement rRT-PCR by providing actionable insights into lineage dynamics and emerging sequence variation. Molecular surveillance using NGS is particularly important for identifying variants that may affect the primer and probe binding regions of established rRT-PCR assays. This highlights the need to routinely monitor and update primer and probe sequences to maintain adequate assay coverage [17]. For example, molecular surveillance has recently identified a three uracil-to-cytosine (3UC) nucleotide substitution in the reverse primer binding site of the US CDC assay [13]. As a result, an additional primer was included to ensure detection of 3UC MeV variants [17].

However, integrating NGS into routine clinical measles diagnostics remains challenging. Limitations include high per-sample costs, the need for specialized laboratory infrastructure, reliance on complex bioinformatics pipelines, variable reference genome quality, and lack of standardized workflows across laboratories [32,33]. Moreover, clear clinical guidelines for when NGS should be used for MeV testing are limited, underscoring the need for diagnostic stewardship and evidence-based incorporation into surveillance strategies [34,35]. In resource-limited countries, the routine use of NGS for pathogen surveillance faces additional barriers, including dependence on external funding, supply chain disruptions, a shortage of trained personnel, and inadequate quality assurance systems [36]. To address these barriers, country-specific approaches, like focusing on high-priority pathogens and outsourcing, can help improve regional pathogen surveillance efforts [37].

### 4.3. CRISPR-Based Diagnostics

CRISPR-based diagnostic platforms use programmable guide RNAs and Cas enzymes to detect target nucleic acids with high specificity and sensitivity [38,39]. Upon recognition of a target sequence, Cas-mediated cleavage generates a detectable signal, enabling rapid and low-cost molecular detection with performance approaching that of PCR [40,41].

For measles diagnosis, CRISPR–Cas assays can be designed to target conserved regions of the MeV genome, enabling fast and specific detection. Their single-nucleotide resolution also makes them well-suited for distinguishing vaccine-derived strains from wild-type virus or for tracking mutations relevant to molecular epidemiology [4,42]. The ease of redesigning guide RNAs could additionally support rapid assay adaptation during outbreaks or the emergence of new MeV variants. CRISPR diagnostics offer greater specificity than rRT-PCR because of an extra nuclease confirmation step and the ability to discriminate at the single-nucleotide level [4,42]. In addition, CRISPR requires less turnaround time and molecular biology expertise, yet still provides technical performance similar to rRT-PCR (Table 1).

Despite these strengths, several barriers currently limit the widespread use of CRISPR diagnostics for measles. Sequence mismatches between gRNAs and target regions can reduce assay sensitivity and standardized protocols, validation studies, and quality assurance frameworks remain insufficient [4,42]. Clinical standardization and regulatory challenges can also limit near-term clinical adoption, which may affect assay performance, inter-laboratory reproducibility and faster access [43]. Furthermore, most CRISPR-based assays still rely on upstream nucleic acid amplification to achieve clinically relevant sensitivity [4]. This requirement increases workflow complexity and dependence on laboratory infrastructure, trained personnel, and extended turnaround times, thereby limiting their suitability as true point-of-care tests [4]. As a result, its applicability in outbreak settings is constrained, where rapid, decentralized testing is essential to support a timely public health response and infection control.

## 5. Conclusions

**Figure 1:**
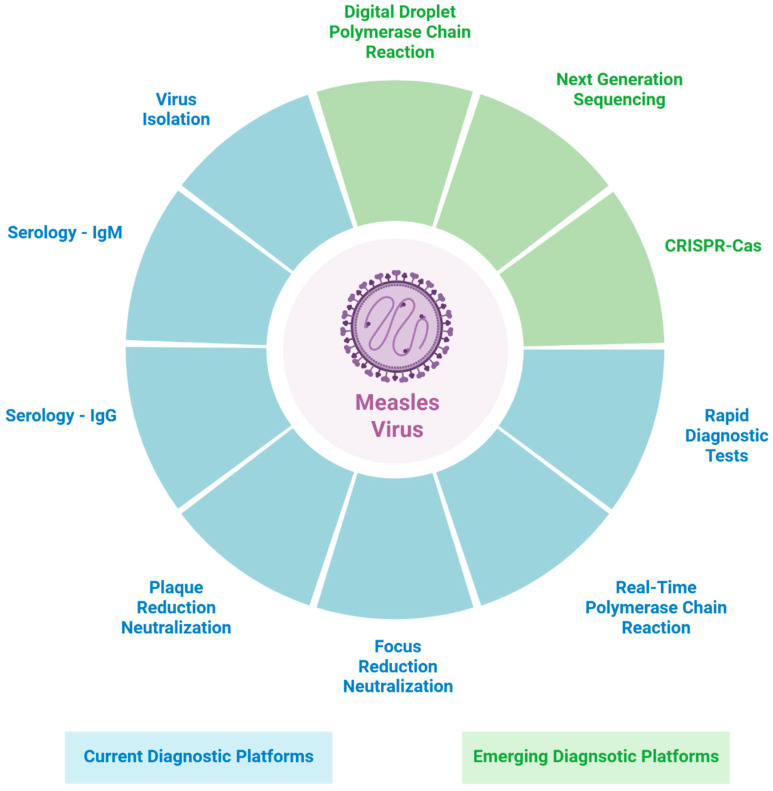
Diagram summary of current and emerging diagnostic platforms for measles virus detection. Figure 1 was created in BioRender. Xue, Y. (2025) https://BioRender.com/sb70pif (accessed on 10 December 2025).

Timely and accurate detection of MeV remains essential for effective clinical management, surveillance, and outbreak control, particularly as global measles activity continues to rise due to persistent gaps in vaccination coverage. Traditional diagnostic approaches, including serology and rRT-PCR, remain the cornerstone of measles testing; however, each has inherent limitations related to sensitivity, specificity, operational complexity, and scalability (Figure 1; Table 1).

Emerging technologies such as ddPCR, NGS, and CRISPR-based assays offer significant potential to enhance analytical sensitivity, enable detailed genomic characterization, and, in some cases, support more decentralized or point-of-care testing (Figure 1). Despite these advantages, their integration into routine measles diagnostics is constrained by high costs, limited standardization, technical requirements, and the absence of robust, large-scale clinical validation. Continued development and harmonization of these technologies will be critical for strengthening measles preparedness and improving global diagnostic capacity.

## Figures and Tables

**Table 1:** Performance comparison of measles virus detection methods.

Method	Target	Sensitivity (%)	Specificity (%)	Technical Requirements	Time To Result
Serology–EIA	IgM/IgG antibodies	75–98	87–99	Specialized virology expertise	3–5 days
Serology-CLIA	IgM/IgG antibodies	94–97	95–98	Automated platform training	0.5–1 h
PRNT	Neutralizing antibodies	98–100	99–100	Specialized virology expertise	3–5 days
FRNT	Neutralizing antibodies	95–100	98–100	Specialized virology expertise	2–3 days
rRT-PCR	RNA	94–99	99–100	Molecular biology expertise or automated platform training	2–6 h
RDT	IgM antibodies	90–95	94–96	Minimal training	0.5 h
ddPCR	RNA	95–100	98–100	Molecular biology and dPCR expertise	4–6 h
NGS	RNA	70–100	90–100	NGS and bioinformatics expertise	6 h–5 days
CRISPR	RNA	96	100	Molecular biology expertise	0.5–1 h

## Data Availability

Not applicable.

## Abbreviations

The following abbreviations are used in this manuscript:

BSL-2	Biosafety level 2
CLIA	Chemiluminescent immunoassay
CSF	Cerebrospinal fluid
ddPCR	Digital droplet polymerase chain reaction
EIA	Enzyme immunoassay
FRNT	Focus reduction neutralization test
LOD	Limit of detection
MeV	Measles virus
N	Nucleocapsid protein
NGS	Next-generation sequencing
PRNT	Plaque reduction neutralization test
RDT	Rapid diagnostic test
rRT-PCR	Real-time reverse transcriptase polymerase chain reaction
SSPE	Subacute sclerosing panencephalitis
US CDC	U.S. Centers for Disease Control and Prevention
WHO	World Health Organization

## References

[1] Dunn J.J., Baldanti F., Puchhammer E., Panning M., Perez O., Harvala H., Practice Pan American Society for Clinical Virology Clinical, Committee Public Policy, Committee the European Society for Clinical Virology Executive (2020). Measles is Back—Considerations for Laboratory Diagnosis. J. Clin. Virol..

[2] Do L.A.H., Mulholland K. (2025). Measles 2025. N. Engl. J. Med..

[3] Ferren M., Horvat B., Mathieu C. (2019). Measles Encephalitis: Towards New Therapeutics. Viruses.

[4] Sharma S., Pokharel Y.R. (2025). Measles and rubella: From Global Health Challenges to Advancements in Molecular Diagnostics in the Elimination Era. Mol. Ther. Nucleic Acids.

[5] Zubach V., Beirnes J., Hayes S., Severini A., Hiebert J. (2024). Diagnostic Accuracy of Commercially Available Serological Tests for the Detection of Measles and Rubella Viruses: A Systematic Review and Meta-Analysis. J. Clin. Microbiol..

[6] US Centers for Disease Control and Prevention: Laborartory Testing for Measles. https://www.cdc.gov/measles/php/laboratories/index.html.

[7] World Health Organization (2018). Manual for the Laboratory-Based Surveillance of Measles, Rubella, and Congenital Rubella Syndrome.

[8] Fappani C., Gori M., Canuti M., Terraneo M., Colzani D., Tanzi E., Amendola A., Bianchi S. (2022). Breakthrough Infections: A Challenge Towards Measles Elimination?. Microorganisms.

[9] Albrecht P., Herrmann K., Burns G.R. (1981). Role of Virus Strain in Conventional and Enhanced Measles Plaque Neutralization Test. J. Virol. Methods.

[10] Vaidya S.R., Kumbhar N.S., Bhide V.S. (2014). Detection of Measles, Mumps and Rubella Viruses by Immuno-Colorimetric Assay and Its Application in Focus Reduction Neutralization Tests. Microbiol. Immunol..

[11] Terletskaia-Ladwig E., Enders G., Meier S., Dietz K., Enders M. (2011). Development and Evaluation of an Automatable Focus Reduction Neutralisation Test for the Detection of Measles Virus Antibodies Using Imaging Analysis. J. Virol. Methods.

[12] Bankamp B., Takeda M., Zhang Y., Xu W., Rota P.A. (2011). Genetic Characterization of Measles Vaccine Strains. J. Infect. Dis..

[13] Perez-Rodriguez F.J., Cherpillod P., Thomasson V., Vetter P., Schibler M. (2024). Identification of a Measles Variant Displaying Mutations Impacting Molecular Diagnostics, Geneva, Switzerland, 2023. Eurosurveillance.

[14] Roy F., Mendoza L., Hiebert J., McNall R.J., Bankamp B., Connolly S., Ludde A., Friedrich N., Mankertz A., Rota P.A. (2017). Rapid Identification of Measles Virus Vaccine Genotype by Real-Time PCR. J. Clin. Microbiol..

[15] Alves A.D.R., Raposo J.V., de Sousa R.M.P., Cardoso C.A.A., Costa P., Araujo J.M., Barreiro S.T.A., Bressan C.D.S., Calvet G.A., de Souza R.V. (2022). Beyond Arboviruses: A Multicenter Study to Evaluate Differential Diagnosis of Rash Diseases and Acute Febrile Illness Cases in Rio de Janeiro, Brazil. PLoS ONE.

[16] Wilson S.E., Zubach V., Lew B., Hasso M., Olsha R., Salvadori M.I., Manoj N., Hiebert J. (2025). Measles Vaccine Virus Mutation Following Vaccination in a Healthy Child Resulting in a False Negative Vaccine Specific PCR Test: Ontario, Canada, 2025. Eurosurveillance.

[17] Beck A.S., Lopareva E.N., Hwang H., Hart D., de Almeida M., Anderson R., Rota P.A., Bankamp B. (2024). Evaluation of the Sensitivity of a Measles Diagnostic Real-Time RT-PCR Assay Incorporating Recently Observed Priming Mismatch Variants, 2024. Eurosurveillance.

[18] Rachlin A., Hampton L.M., Rota P.A., Mulders M.N., Papania M., Goodson J.L., Krause L.K., Hanson M., Osborn J., Kelly-Cirino C. (2024). Use of Measles and Rubella Rapid Diagnostic Tests to Improve Case Detection and Targeting of Vaccinations. Vaccines.

[19] Koczula K.M., Gallotta A. (2016). Lateral Flow Assays. Essays Biochem..

[20] Senin A., Noordin N.M., Sani J.A.M., Mahat D., Donadel M., Scobie H.M., Omar A., Chem Y.K., Zahari M.I., Ismail F. (2024). A Measles IgM Rapid Diagnostic Test to Address Challenges with National Measles Surveillance and Response in Malaysia. PLoS ONE.

[21] Quan P.L., Sauzade M., Brouzes E. (2018). dPCR: A Technology Review. Sensors.

[22] Kojabad A.A., Farzanehpour M., Galeh H.E.G., Dorostkar R., Jafarpour A., Bolandian M., Nodooshan M.M. (2021). Droplet Digital PCR of viral DNA/RNA, Current Progress, Challenges, and Future Perspectives. J. Med. Virol..

[23] Javornik Cregeen S., Tisza M.J., Hanson B., Cook M., Surathu A., Schneider R., Wu J., Short K., Domakonda K., Hopkins L. (2025). Sequencing-Based Detection of Measles in Wastewater: Texas, January 2025. Am. J. Public Health.

[24] Joseph K.M., Chen X., Parikh D., Rios J., Troisi C.L., Tisza M.J., Maresso A.W., Hanson B.M., Gitter A., Deegan J. (2025). Detection of Measles in Texas Wastewater. medRxiv.

[25] Näyhä S. (2005). Environmental temperature and mortality. Int. J. Circumpolar Health.

[26] Wu J., Wang M.X., Kalvapalle P., Nute M., Treangen T.J., Ensor K., Hopkins L., Poretsky R., Stadler L.B. (2024). Multiplexed Detection, Partitioning, and Persistence of Wild-Type and Vaccine Strains of Measles, Mumps, and Rubella Viruses in Wastewater. Environ. Sci. Technol..

[27] Langan L.M., Bain F.L., Snow C.C., Oldfather J., Sagvold O., Kaneshiro K., Nwagwu C., Choi H., Wronski A.R., Alamin M. (2025). Spatially Informed Wastewater Differentiation Among Locations During an Ongoing Measles Outbreak in Texas, USA. ACS Environ. Au.

[28] Gwinn M., MacCannell D., Armstrong G.L. (2019). Next-Generation Sequencing of Infectious Pathogens. JAMA.

[29] NIHR Global Health Research Unit on Genomic Surveillance of AMR (2020). Whole-Genome Sequencing as Part of National and International Surveillance Programmes for Antimicrobial Resistance: A Roadmap. BMJ Glob. Health.

[30] Bankamp B., Kim G., Hart D., Beck A., Ben Mamou M., Penedos A., Zhang Y., Evans R., Rota P.A. (2024). Global Update on Measles Molecular Epidemiology. Vaccines.

[31] Gruber C.E.M., Gioacchini S., Fabeni L., Rueca M., Bordi L., Lalle E., Baggieri M., Bucci P., Fioravanti R., Giuseppetti R. (2025). Molecular Investigation on Measles Cases Rise and Variants Co-Circulation in the Lazio Region, Italy. Virol. J..

[32] Chiu C.Y., Miller S.A. (2019). Clinical Metagenomics. Nat. Rev. Genet..

[33] Liu Y., Ma Y. (2024). Clinical Applications of Metagenomics Next-Generation Sequencing in Infectious Diseases. J. Zhejiang Univ. Sci. B.

[34] Gaston D.C., Chiang A.D., Dee K., Dulek D., Banerjee R., Humphries R.M. (2024). Diagnostic Stewardship for Next-Generation Sequencing Assays in Clinical Microbiology: An Appeal for Thoughtful Collaboration. Clin. Lab. Med..

[35] Gaston D.C. (2023). Clinical Metagenomics for Infectious Diseases: Progress Toward Operational Value. J. Clin. Microbiol..

[36] Getchell M., Wulandari S., de Alwis R., Agoramurthy S., Khoo Y.K., Mak T.M., Moe L., Stona A.C., Pang J., Momin M. (2024). Pathogen Genomic Surveillance Status Among Lower Resource Settings in Asia. Nat. Microbiol..

[37] Pronyk P.M., de Alwis R., Rockett R., Basile K., Boucher Y.F., Pang V., Sessions O., Getchell M., Golubchik T., Lam C. (2023). Advancing Pathogen Genomics in Resource-Limited Settings. Cell Genom..

[38] Kaminski M.M., Abudayyeh O.O., Gootenberg J.S., Zhang F., Collins J.J. (2021). CRISPR-Based Diagnostics. Nat. Biomed. Eng..

[39] Yang H., Zhang Y., Teng X., Hou H., Deng R., Li J. (2023). CRISPR-Based Nucleic Acid Diagnostics for Pathogens. Trends Anal. Chem..

[40] Broughton J.P., Deng X., Yu G., Fasching C.L., Servellita V., Singh J., Miao X., Streithorst J.A., Granados A., Sotomayor-Gonzalez A. (2020). CRISPR-Cas12-Based Detection of SARS-CoV-2. Nat. Biotechnol..

[41] Huang T., Zhang R., Li J. (2023). CRISPR-Cas-Based Techniques for Pathogen Detection: Retrospect, Recent Advances, and Future Perspectives. J. Adv. Res..

[42] Pinchon E., Henry S., Leon F., Fournier-Wirth C., Foulongne V., Cantaloube J.F. (2024). Rapid Detection of Measles Virus Using Reverse Transcriptase/Recombinase Polymerase Amplification Coupled with CRISPR/Cas12a and a Lateral Flow Detection: A Proof-of-Concept Study. Diagnostics.

[43] Rodriguez-Manzano J., Subramaniam S., Uchea C., Szostak-Lipowicz K.M., Freeman J., Rauch M., Tinto H., Zar H.J., D’Alessandro U., Holmes A.H. (2024). Innovative Diagnostic Technologies: Navigating Regulatory Frameworks Through Advances, Challenges, and Future Prospects. Lancet Digit. Health.

